# The Many Faces of Head and Neck Surgery in 2022 and Looking Ahead!

**DOI:** 10.3390/jcm11113174

**Published:** 2022-06-02

**Authors:** Luca Giovanni Locatello, Oreste Gallo

**Affiliations:** 1 Department of Otorhinolaryngology, Sant’Antonio Abate Hospital, Azienda Sanitaria Universitaria Friuli Centrale, 33028 Tolmezzo, Italy; 2 Department of Otorhinolaryngology, Careggi University Hospital, Largo Brambilla 3, 50134 Florence, Italy; oreste.gallo@unifi.it; 3 Department of Clinical and Experimental Medicine, University of Florence, 50139 Firenze, Italy

Head and neck (HN) cancer, which mainly presents in the form of squamous cell carcinoma, was the seventh most common cancer worldwide in 2018, with approximately 890,000 new cases and 450,000 deaths [1]. Its incidence is rising in both less developed countries, due to the increased exposure to classical risk factors (tobacco smoking and alcohol), as well as in high-income nations, due to the spreading of high-risk serotypes of human papillomavirus (HPV-16 and HPV-18) [2]. Survival rates range from 70–80% at five years for early HN cancer (i.e., with neither nodal metastasis at presentation nor evidence of extra-organ extension) to an estimated overall survival of 30–40% at advanced stages [1,2]. Unfortunately, the latter is the most common clinical presentation, and a recent study from the USA showed that the age-adjusted incidence rates for stage IV HN cancer have significantly increased by 26.1% over the last two decades [3].

The first historical report of such a disfiguring disease dates back to the Ancient Egyptians and Greeks (with the first description of a case of oral cancer); it was in the Roman Empire, though, that *Aulus Cornelius Celsus* was credited to have performed the first HN operation in the form of a lip tumor excision [4,5]. Back to the present day, we know that the surgical removal of HN cancer remains a cornerstone of its management, along with chemo-radiation “organ-preserving” strategies [6]. More interestingly, the recent literature suggests that HN surgery must be performed shortly after a cancer diagnosis because time-to-surgery may represent an independent prognostic factor [7].

The field of head and neck surgery has been profoundly revolutionized, like many others, by the technological advances in recent decades, and open, microscopic, endoscopic, and robotic techniques have allowed us to achieve unprecedented results [8,9,10]. Many overlapping areas now exist in the HN surgery discipline, and otolaryngologists must work with (and learn from) plastic surgeons, neurosurgeons, and maxillofacial surgeons. For example, we are now capable of performing a totally endoscopic transnasal removal of sinonasal malignancies, even when invading the dural membrane; we can offer our patients “scarless” transaxillary or transoral thyroidectomy; or we can perform robotic transoral pharyngectomy and partial/total laryngectomies, or even endoscopic-assisted lateral skull base dissections [11,12,13,14,15].

As the boundaries of HN surgery expand, the chance of impairing or even permanently damaging critical structures (cranial nerves, major vessels, brain, etc.) and related physiological functions (swallowing, phonation, sense of smell and taste, etc.) increase [16]. The intratemporal and extratemporal facial nerves during otological and parotid surgery, and the superior and inferior laryngeal nerves during thyroid and parathyroid surgery, respectively, are well-known anatomical examples of this. The extensive demolition of oral, pharyngeal, and sinonasal structures instead requires a fine application of regional or free flaps in order to at least partially regain the functionality of the upper aerodigestive tract. Sometimes, and despite all efforts, permanent tracheostomy and gastrostomy tube dependency rates are non-negligible (up to 64% in some series), a fact which is unavoidably associated with a poor quality of life [17,18]. There are some surgical complications that we must prevent and manage, sometimes by applying classical techniques: for instance, in transoral robotic surgery, bleeding is a very common event, and the transcervical ligation of the lingual artery has been shown to reduce the risk of fatal hemorrhage but not its incidence [19]. For skull base tumors, instead, the preoperative embolization of the internal carotid artery along with a deep knowledge of the microscopic and endoscopic landmarks now permit a safe dissection for previously considered unresectable tumors (e.g., endoscopic nasopharyngectomy operations for post-RT nasopharyngeal carcinoma) [20].

Many patient-related predictive factors of complications have been identified, such as age or the presence of comorbidities; on the other hand, because HN surgery is being more commonly performed as a salvage strategy, we tend to operate on previously irradiated patients who are, by definition, fragile [21]. Preoperative screening for patients who are most at risk and a rigorous surgical technique are the mainstay for reducing these complications, which can sometimes be managed in a conservative (medical) manner [22]. In conclusion, the prognosis of HN cancer has not improved by a large extent in the last century, and if we exclude oropharyngeal HPV-related cases, a little over half of the patients who are diagnosed with this disease will survive beyond 5 years. As we are continuously refining our diagnostic and therapeutic approaches to HN disorders, the aforementioned risks and complications are, and will always be, present. Optimistically, we believe that by following sound evidence-based clinical recommendations, and thanks to the diffusion of novel technologies, devices, and surgical expertise, the incidence of these adverse events will be brought nearly to zero. As Guest Editors of this Special Issue, we would like to conclude this Editorial by thanking the authors who have submitted their excellent papers so far, the reviewers for their punctual remarks, and all the staff of the *Journal of Clinical Medicine* for their constant support.
